# The Development of Bilateral Mastoiditis Following Acute Otitis Media in a Pediatric Patient with Limited Access to Health Care

**DOI:** 10.7759/cureus.35113

**Published:** 2023-02-17

**Authors:** Marta Telatin, Dylan S Irvine, Marc M Kesselman, Joshua M Cullen

**Affiliations:** 1 Medical School, Nova Southeastern University, Dr. Kiran C. Patel College of Osteopathic Medicine, Davie, USA; 2 Rheumatology, Nova Southeastern University, Dr. Kiran C. Patel College of Osteopathic Medicine, Davie, USA; 3 Family and Community Medicine, Community Health Center of West Palm Beach, West Palm Beach, USA

**Keywords:** inpatient pediatrics, pediatric preventative medicine, otitis media complication, acute mastoiditis, pediatrics education

## Abstract

Infection of the mastoid cells, known as mastoiditis, can develop due to untreated otitis media, in which bacteria colonize the mastoid air cells that line the inner and middle ear. Antibiotic therapy for otitis media has made the development of mastoiditis a very rare occurrence. However, despite its low prevalence, it is important to keep this complication in mind when treating otitis media in the pediatric population due to the increased susceptibility of mastoiditis in this demographic. Furthermore, pediatric patients of lower socioeconomic status who have limited access to health care may be at an even greater risk for the development of mastoiditis. We report a case of a pediatric patient with significant barriers to health care who developed bilateral mastoiditis as a complication of otitis media, requiring hospitalization and intravenous antibiotic therapy. The patient also experienced hearing loss as a sequela of the infection. Improved access to medical care, parent or guardian education on how to recognize primary otitis media infections, and the use of adequate antibiotic therapy when indicated can effectively prevent the development of mastoiditis following otitis media infections among patients.

## Introduction

Bilateral mastoiditis is a rare complication of otitis media, occurring at a prevalence rate of approximately 0.0018% among the pediatric population [1]. Mastoiditis is an infection of the mastoid air cells within the temporal bone that is preventable with rapid and effective treatment of otitis media for patients who have access to basic health care [2]. Prior to the development of antibiotics, mastoiditis was a common cause of death in children [3]. During the pre-antibiotic era, around 20% of cases of otitis media developed into mastoiditis [4]. Key indicators of mastoiditis include redness and swelling behind the ear causing the auricle to bulge. The child may also complain of hearing loss, headache, or ear pain. The current gold standard diagnosis of mastoiditis is a computerized tomography (CT) scan, demonstrating opacification of mastoid air cells, mastoid cortex destruction, and erosion of cell septum [5]. Sensorineural hearing loss can occur consequently to mastoiditis, as was the case in our patient [6]. The long-term hearing sequela following a mastoiditis infection are still unclear, and further research studying this relationship is required. Proper education and prevention of the possible complications of untreated otitis media are crucial in the prevention of bilateral mastoiditis, especially in underserved patients with limited access to health care.

## Case presentation

We report the case of a nine-year-old male patient who presented to a health clinic for a follow-up regarding residual hearing deficits and vertigo following hospitalization for bilateral otomastoiditis. The patient and his family recently immigrated to the United States from South America. This difficult transition created both socioeconomic and language barriers preventing the patient from obtaining timely access to health care. The patient’s guardian provided the history of his present illness. Prior to seeking medical attention, the patient complained of experiencing bilateral hearing loss, ear pain, and vertigo for 10 days. No supportive care was administered by the patient’s guardian during this time due to their uncertainty about how to effectively treat his symptoms. The patient had no significant medical history and did not take any prescription medications. 

As the patient’s symptoms continued to deteriorate with worsening ear pain, nausea, and vertigo, the family sought intensive care for the child in the emergency department. On his first visit to the emergency department, the patient was found to have what appeared to be an ear infection. The patient was prescribed ear drops and sent home. He did not find relief from the ear drops, and his symptoms continued to worsen. Subsequently, he began to have recurrent daily episodes of hematemesis and spiking fevers. Another five days passed, and the child continued to show no signs of improvement. The patient was then brought to a separate hospital and was admitted as he was noted to appear toxic. On admission, the patient had a temperature of 100.6 ° Fahrenheit, a heart rate of 116 beats per minute, and a blood pressure of 96/58 mmHg. The patient weighed 24 kilograms and measured 120 centimeters in height.

Upon physical examination, adenoidal hypertrophy was noted. Furthermore, the right tympanic membrane was intact, thickened, and opaque. The right middle ear showed a white, purulent appearing effusion. The left tympanic membrane was unable to be visualized. The left middle ear was unable to be visualized. Blood work revealed elevated white blood cells, c-reactive protein, lactate dehydrogenase, and neutrophil count. The laboratory results are shown in Table 1. These elevated biomarkers indicated a high suspicion of an active and progressing infection in the patient. 

**Table 1:** Summary of Complete Blood Count (CBC), C-Reactive Protein (CRP), and Lactate Dehydrogenase (LDH) Laboratory Results.

Diagnostic Laboratory Test	Value	Reference Range
White Blood Cell Count	16.1 x 10^3^/mcL	5.0-14.5 x10^3^/mcL
Neutrophil Relative	88.4%	33.0-70.0%
Neutrophil Absolute	14.2 x 10^3^/mcL	1.5-6.4 x10^3^/mcL
CRP Quant	18.5 mg/dL	0.1-0.4 mg/dL
LDH	263.0 units/L	135.0-225.0 units/L

**Figure 1:**
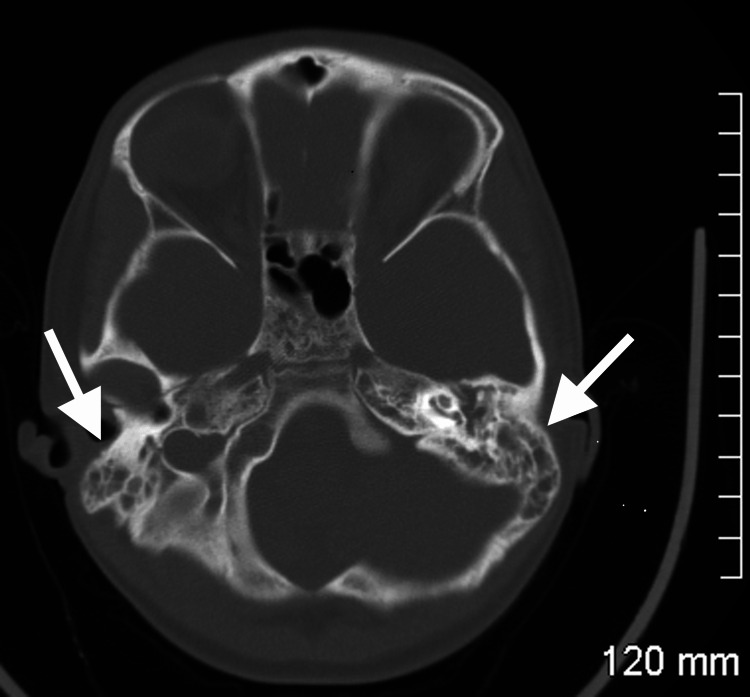
CT scan with visualization of bilateral otomastoiditis. CT: Computerized tomography

The patient underwent a CT scan of the head with and without contrast. The CT scan demonstrated moderate mucosal thickening of the maxillary ethmoid and right sphenoid sinuses with bilateral extensive opacification of the mastoid air cells and middle ears, which is consistent with a diagnosis of bilateral otomastoiditis. Visualization of the CT scan can be seen in Figure 1. The patient was started on intravenous (IV) vancomycin and ceftazidime.

After the CT scan was done, a lumbar puncture was performed, and spinal fluid was sent for analysis to rule out meningitis. Results of the spinal fluid analysis are shown in Table 2 and indicated that there was not a concurrent infection of the meninges. A negative purified protein derivative test was obtained to rule out tuberculosis. 

**Table 2:** Results of Spinal Fluid Analysis.

Spinal Fluid Analysis	
Total Volume Cerebral Spinal Fluid (CSF)	1 mL
Appearance Unspun	Clear
Color Unspun	Colorless
Reb Blood Cell CSF	10/mm3
White Blood Cell CSF	2/mm3
Lymphocyte CSF	44%
Monocyte CSF	52%
Macrophage CSF	4%

Antibiotics are the key treatment for mastoiditis; however, further procedures including myringotomy, tympanostomy tube placement, and mastoidectomy may be necessary for complicated cases of mastoiditis [5]. Among the pathogens that are associated with mastoiditis, the most common microbe responsible is *Streptococcus pneumoniae* [7]. There are several life-threatening complications of mastoiditis, ranging from intracranial subdural abscess to extracranial subperiosteal abscess. IV antibiotics such as ceftriaxone are the standard of care for uncomplicated mastoiditis [8]. It is important to note that a repeat CT scan must be performed prior to surgical intervention. The patient responded to IV treatment of ceftriaxone and vancomycin within 48 hours. Thus, it was deemed surgical intervention was not necessary. Twenty-four hours after initiation of IV antibiotics, the fevers and emesis resolved. The patient was treated with IV antibiotics for 10 days. There were no side effects associated with the treatment. Blood cultures were collected on the first day of admission and demonstrated no growth during a five-day incubation period. The patient was discharged and prescribed an additional 10-day course of oral linezolid and cefdinir for continuation of care at home. 

## Discussion

The development of bilateral mastoiditis as a complication of otitis media has become a relatively preventable pathology. However, despite its relatively low prevalence, mastoiditis is a possible complication of otitis media that physicians must keep in mind when treating pediatric populations to avoid detrimental long-term sequala. This case highlights how treatment protocols must be tailored to the individual patient, as given recommendations may not take into consideration limitations of care that the patient may face. Limitations of care may include language barriers, socioeconomic barriers, lack of transportation, and decreased knowledge of infectious pathology. 

Guidelines set forth by the American Academy of Pediatrics (AAP) and American Academy of Family Physicians (AAFP) suggest that initial observation and delay of treatment of acute otitis media in an uncomplicated pediatric patient may be beneficial to decrease undesirable side effects associated with antibiotic use [9]. Immediate initiation of treatment is suggested in pediatric patients at increased risk for severe complications, including infants <six months of age, patients who are immunocompromised, toxic appearing, or have craniofacial abnormalities (e.g., cleft palate) [9]. However, suggestions on treatment for pediatric patients of lower socioeconomic status (SES) with lack of access to health care were not discussed. Widespread research has shown that there is an association between low SES and poor health outcomes [10]. Thus, immediate treatment with antibiotics for acute otitis media could possibly be considered for patients of lower SES if the physician suspects barriers to following up to seek further treatment if cases progress to being more severe. 

As demonstrated by this case report, individuals of lower SES who have limited access to consistent health care can be at increased risk for the development of complications from acute infectious diseases [11]. Thus, it is vital that patients are evaluated on a case-by-case basis. The patient presented here developed mastoiditis after the initial treatment of topical antibiotics was not sufficient to eradicate the infection. The patient's symptoms continued to worsen; however, the family did not have access to primary health care to have their child revaluated leading to the development of mastoiditis. Also, the family members may not have been educated about the signs and symptoms of otitis media in children and thus may not have suspected this pathology from its onset, possibly delaying the patient’s time from symptom onset to medical intervention. Furthermore, the delay in treatment of this patient ultimately resulted in the need for hospitalization to successfully treat this complication which can place a determinantal burden on both the family and the health care system. These points emphasize the importance of secondary prevention measures taken to prevent the development of complications from simple otitis media infections. Such measures may include increasing access to primary care visits among individuals with limited access to health care and placing an emphasis on educating parents of young children during annual well-child visits, rather than fighting an infection once it has developed. These measures could be effective in preventing the development of mastoiditis, among other infections with serious morbidities, within the pediatric population. 

## Conclusions

Here, we have reported a case of bilateral mastoiditis, an extremely rare complication of otitis media. Pediatric patients, especially those of lower SESs or those with limited access to health care, are at increased risk of the development of mastoiditis and the potential sequela that can arise from the progression of otitis media. Mastoiditis is diagnosed clinically with confirmation using CT imaging and antibiotics are the first line of treatment. However, if significant progression of the infection has occurred, invasive treatments such as surgery may be necessary. Emphasizing the importance of secondary disease prevention and early intervention in at-risk pediatric populations is critical to prevent complications of otitis media, such as mastoiditis.
